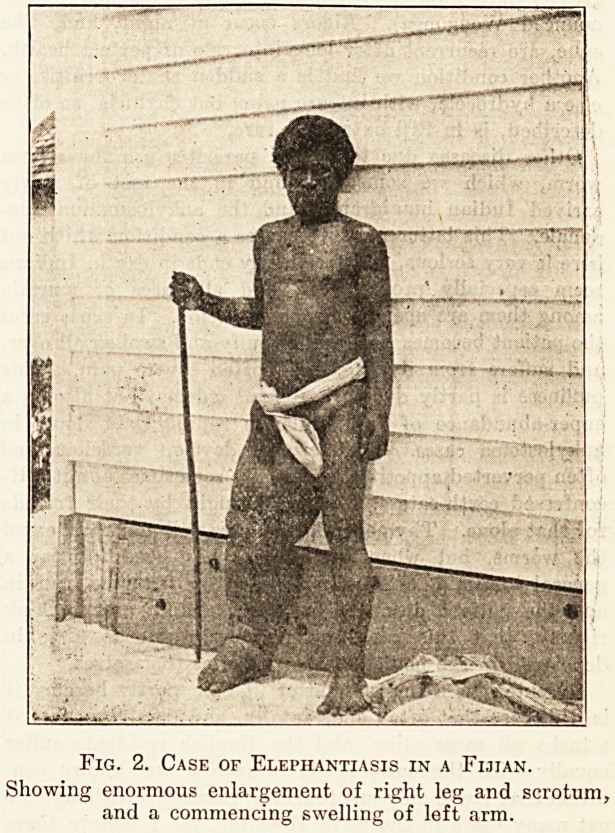# Fiji: Its Hospitals and Diseases—II

**Published:** 1908-04-18

**Authors:** T. R. St. Johnston

**Affiliations:** Resident Medical Superintendent, Colonial Hospital, Suva.


### HOSPITAL ADMINISTRATION.

### CONSTRUCTION AND ECONOMICS.

### FIJI: ITS HOSPITALS AND DISEASES.—II.

By T. R. St. JOHNSTON, M.R.C.S., L.R.C.P., M.S.A., Resident Medical Superintendent, Colonial Hospital, Suva.

I have previously described a Fijian hospital and some of the patients to be found there. It may be of interest to see what unusual diseases they suffer from, and what native drugs and remedies are known to them.

Frambœsia, or yaws, by its universal prevalence, has claim to the first place on the list, and as this is a disease seldom or never seen in temperate climates, it is of considerable interest to those unacquainted with tropical medicine. Like syphilis, it is divided into three stages—a primary or constitutional one, with fever and general pains; a secondary or furfuraceous stage, with desquamation of the skin (often overlooked), and a tertiary or true yaw stage, where the papules, often an inch in diameter, develop and are filled with a yellow cheese-like material, which later on turns green, but never breaks down to pus. If properly treated, that is by means of iodides and mercury ointments locally, the yaw soon dries up and the crust falls off; but if left alone it often develops more deeply, swellings not unlike the gummata of syphilis arise, and eventually break down to form large ulcers, which again fortunately respond well to potassium iodide. The commonest situations are the chest, face, and limbs; while frequently a yaws ulcer occurs in the soft palate,

Fig. 1. Case of Yaws in a Punjab Indian.
Showing tumours the size of a raspberry on the face and chest, the favourite situations.

causing a perforation for all the world like syphilis. There is also a general periostitis of the bones, with tenderness, which frequently develops in old Frambœsia patients, with no other signs.

All these occurrences are so much like syphilis that many observers have doubted the existence of a separate disease, but I cannot too strongly emphasise the fact that here in Fiji, where syphilis—among Fijians—is practically unknown, we have striking occular demonstration of the pre-

Fig. 2. Case of Elephantiasis in a Fijian.
Showing enormous enlargement of right leg and scrotum, and a commencing swelling of left arm.

sence of a distinct disease; quite apart from the bacteriological proof afforded by the presence of Castellani's spirochaete, which, though much like that of syphilis, has several points of differentiation.

Yaws is so common in Fiji—(coko, pronounced thoko, it is called)—that a few years ago every Fijian was said to have had it at some time in his life, and it was even customary to inoculate the children with it, as a mild attack was considered, and really is, a preventive of other and worse attacks. Now the Indian immigrants are taking it badly, and call it among themselves "Fiji bimari," or the "Fijian sickness," but curiously enough Polynesians very seldom acquire it. Patients here are isolated when possible, and there is a special "yaws block" away from the other buildings for the housing of them, and owing to this and other precautionary measures in the country districts the disease is being slowly but surely stamped out.

The second great disease among natives in Fiji is elephantiasis, that extraordinary and often ludicrous condition where apparently a pigmy man is attached to some giant's limb rather than the limb attached to the man. Though we seldom, or never, find the Filaria Bancrofti in the blood of patients here, yet there is no doubt that this is the origin of the trouble, and that the mosquito is the intermediary host. The only strange thing is that though mosquitoes (the Culices) abound, and white people suffer from their bites even more than the natives, yet elephantiasis is exceedingly rarely found among Europeans.

Besides the enormous swellings of legs or scrotum, pictures of which are so common in text-books, called by the Fijians "Tauna," we also find some interesting conditions not generally known, such as acute lymphangitis of one particular gland or set of glands, as the epi-trochlear, accompanied by a high temperature often shooting up to 105° or more in a few hours, and great pain. This condition they name "Waqaqa" (pronounced Wongonga). Rigors occur at night, and, like ague, are recurrent after long intervals of perfect health. Another condition we find is a sudden acute orchitis, or else a hydrocele, with intense pain; but chyluria, so often described, is in Fiji extremely rare.

Other diseases due to animal parasites are the guinea worm, which we sometimes find in the feet of newly arrived Indian immigrants, and the ankylostomum duodenale. This latter parasite causes a condition which out here is very serious, and frequently ends in death. Indians seem especially prone to it, and all cases of anæmia among them are open to strong suspicion. In acute cases the patient becomes remarkably puffy and swollen all over, and suffers from dyspnœa and often severe pain. This puffiness is partly due to a general œdema, but also to a super-abundance of fat, which is rapidly laid down in ankylostoma cases. The patients develop voracious and often perverted appetites, so that we sometimes admit self-confessed earth-eaters, who are brought by their friends for that alone. Thymol in large doses will generally expel the worms, but when the disease has gone too far a general toxæmia, often accompanied by jaundice, sets in and the patient dies. It has been stated, and I think rightly, that this condition is largely due to a toxin developed in the bodies of the parasites themselves.

Dysentery is a serious matter in Fiji, partly because it is a disease that often ends fatally, and partly because it attacks all races alike, and the English residents suffer equally with the natives. It is such a well-known condition that I will only mention it in passing. Although we get many cases in the Suva Hospital, yet I believe there are more, relatively to the population, to be found in the hospitals situated in the country districts, probably due to the fact that the water supply, though good throughout Fiji, is exceptionally good in the capital.

In connection with dysentery one should mention tropical abscess, so often a direct sequel of an attack of the former disease. This is a common trouble, particularly among Europeans, and usually requires surgical interference, though if the general hepatitis be diagnosed early enough, I find that any breaking down can often be averted by large doses of ammonium chloride.

Dengue fever occurs in epidemics, and at the time of writing we have just recovered from an outbreak of it only to find a mild but quite distinct epidemic of simple influenza succeeding it. Malaria, on the other hand, is not found in Fiji, except for occasional cases introduced from neighbouring Pacific Islands. How long we shall enjoy such immunity it is impossible to say, but should the Anopheles ever be introduced here, or even should the Culices that we possess take on malaria carrying functions, as suggested by Sir Patrick Manson, it will be a great blow to the Colony.

Leprosy is, perhaps, the most serious problem, and every effort is now being made to exterminate it. For many years there has been a leper station on the island of 'Beqa (Mbenga), just off the mainland, but residence there has been only voluntary, except in special instances where compulsion may be used. But there has just been drafted through the Colonial Parliament a Bill which will reorganise the present system, and in future every leper in the colony will be sent to this segregation island. I have heard that the leaves of the "totodra," a small native flower rather like a violet, are taken for this disease, but it is difficult to be accurate about native remedies, as the people are extremely reticent about their herbs and medicines.

But I have had proofs that the barks of two trees, the tavolo and the mulo-mulo, when made into infusions have remarkable antiseptic properties, and are used among other things in the treatment of thrush, while the milky juice of the lolo, a sort of mulberry tree, is employed for its styptic effect. Also the 'bovo leaves are said to reduce a high temperature, and the hibiscus is taken as an aperient.

The most curious instance of native remedies is the operation of "cokalosi," which consists of passing a grooved wooden staff down the urethra, and cutting on to it for a distance of some three inches, allowing the bleeding to go on till it either ceases of its own accord, or—as is not unknown—the patient dies. This barbarous old custom, now seldom seen, was formerly performed with much ceremony under the impression that when a man was ill all the bad blood, and the evil spirits too, collected in these regions, and must be let free.

Another surgical remedy found in Fiji is the cutting with sharp shells wherever there is a pain. There may be an abscess, in which case the free incision does good; but quite as often there is no justification for the procedure, and we sometimes have patients in hospital covered with big keloid scars raised well up from the surface, where this cutting has been performed. I have seen the same thing among the Red Indians around the Rocky Mountains, and believe it is done with the same idea.

These few instances of diseases and remedies in Fiji that I have been able to gather together are all found in far greater numbers out in the provincial hospitals, where the country is still to a large extent uncivilised, and often the doctor in charge is the only white man for many miles in every direction. Wild out-of-the-way places many of these are, yet the hospitals are necessary, and nurses are out of the question, so that the doctor—with the help of one or two native practitioners—has to do not only the treating but also the nursing of patients from a district which is sometimes nearly half as big as Jamaica! But the natives are, on the whole, a healthy race and are always cheerful and obliging, so that half the battle is won before the doctor appears on the scene. As the land is taken up and settled on, and as commerce spreads its way, so will the provincial hospitals and their accommodation have to be increased. At present there are about a dozen or more hospitals scattered through the islands, of which some four or five are kept by the great sugar companies which support the staple industry of the colony, but the doctors to these latter hospitals are all Government medical officers, who attend to the plantations under an agreement between the Government and the sugar companies.

There is plenty of medical work in Fiji, and the eighteen officers in the medical service are none too many; while as the place increases in prosperity, as it is doing now, more will probably be required, and also obtained, for the pay is good, and there is a pension to look forward to. The isolation is a drawback, but the climate is healthy and far removed from the common notion of the "dangerous tropical countries." In fact, as Kipling has said, these South Pacific islands are truly "the Islands of the Blest."

## Figures and Tables

**Fig. 1. f1:**
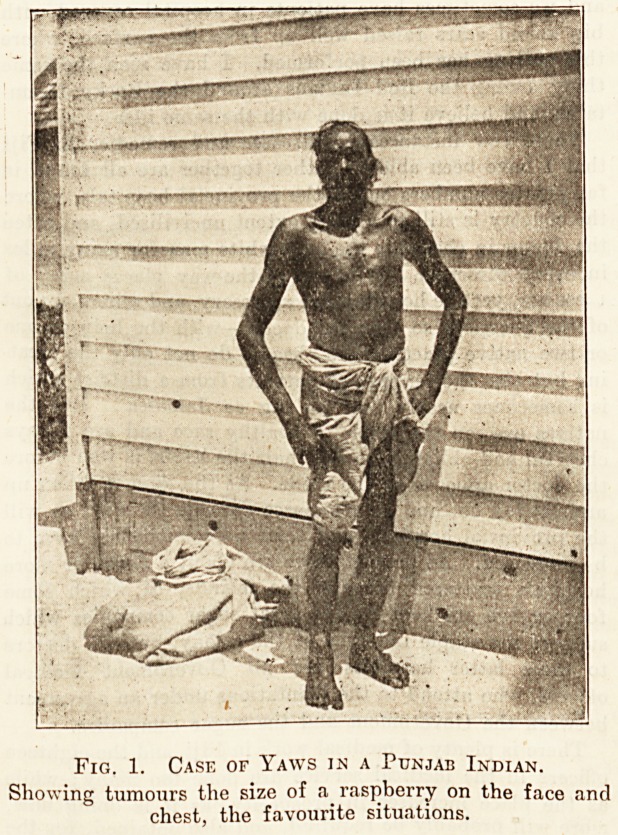


**Fig. 2. f2:**